# Performance Modulation of S-CO_2_ Brayton Cycle for Marine Low-Speed Diesel Engine Flue Gas Waste Heat Recovery Based on MOGA

**DOI:** 10.3390/e24111544

**Published:** 2022-10-27

**Authors:** Liangtao Xie, Jianguo Yang

**Affiliations:** 1 School of Naval Architecture, Ocean and Energy Power Engineering, Wuhan University of Technology, Wuhan 430063, China; 2 Key Laboratory of Marine Power Engineering Technology Transportation Industry, Wuhan 430063, China; 3 National Engineering Laboratory for Marine and Ocean Engineering Power System, Electronic Control Sub-Laboratory for Low-Speed Engine, Wuhan 430063, China

**Keywords:** marine low-speed diesel engine, waste heat recovery of flue gas, S-CO_2_ recompression Brayton cycle, waste heat modulation, thermodynamic performance, the exergy analysis

## Abstract

(1) Background: the shipping industry forced ships to adopt new energy-saving technologies to improve energy efficiency. With the timing modulation for the marine low-speed diesel engine S-CO_2_ Brayton cycle, the waste heat recovery system is optimized to improve fuel economy. (2) Methods: with the 6EX340EF marine low-speed diesel engine established in AVL Cruise M and verified by the bench test data, the model of the S-CO_2_ Recompression Brayton Cycle (SCRBC) system for the low-speed engine flue gas waste heat recovery was developed in EBSILON, and verified by SANDIA experimental data. On this basis, the effects of injection timing and valve timing parameters on the comprehensive performance of the main engine and the waste heat recovery system were investigated. By optimizing the timing modulation parameters through multi-objective genetic algorithm (MOGA) and evaluating the flue gas waste heat recovery from the perspective of thermodynamic performance and emission reduction, the research on the performance modulation method of the S-CO_2_ Brayton Cycle for flue gas waste heat in marine low-speed engines has been completed. (3) Results: the SCRBC with waste heat modulation will further increase the total power and efficiency, which in turn brings about a reduction in the fuel consumption rate. The efficiency of the SCRBC system with the addition of waste heat modulation increases by 2.28%, 1.04% and 2.07% at 50%, 75% and 100%, respectively. After adding the residual heat modulation, the maximum annual CO_2_ emission reduction of 748.51 × 10^3^ kg·a^−1^ occurred at 50% load; with the exergy analysis, the cooler has the largest system exergy loss of 165 kW, with the exergy loss efficiency of 2.06% under 100% load. (4) Conclusions: the research on the performance modulation method of S-CO_2_ Brayton cycle for flue gas waste heat in the marine low-speed engine has been completed, which further improves the efficiency of the system and can be extended to other engines.

## 1. Introduction

The shipping industry plays a pivotal role in the growth of the global economy, and the International Maritime Organization’s (IMO) new CO_2_ emission regulations are forcing ships to adopt new energy-saving technologies to improve their energy efficiency [1]. As the primary power source for ocean shipping, the marine low-speed diesel engine waste heat recovery (WHR) is one of the key technologies to reduce CO_2_ emissions and lower the energy efficiency design index (EEDI) of ships [2]. Marine low-speed diesel engine waste heat presents various forms of distribution, with the characteristics of multi-grade, large temperature difference waste heat [3]. At present, the most advanced high-power marine low-speed two-stroke diesel engine thermal efficiency has been closed to 55%, and the exhaust temperature after the turbocharger is generally in the range of 230–250 °C [4]. The heat loss taken away by the flue gas is the most extensive part, accounting for 22–30% of the input heat, but still has a high waste heat potential.

At present, the utilization of flue gas waste heat from marine low-speed diesel engines mainly includes power turbine (PT) [5,6], electrical turbine (ET) [7], thermoelectric generator (TEG) technology [8,9] and organic Rankine cycle (ORC) [10,11,12], etc. PT/ET is a traditional turbine system solution to effectively recover the exhaust energy of ships, but the efficiency is not high and the structure is not compact; TEG is often applied to low-grade heat sources such as cylinder liner cooling water, and more applications are found in car engines. The maximum circulation temperature of ORC is constrained by the thermal stability of organic work medium. The ammonia–water mixtures are used as work mediums to change the fixed temperature characteristics in KC, but also only for low-grade heat sources. Supercritical carbon dioxide (S-CO_2_) Brayton cycle power generation is characterized by high efficiency, high power density and small size. It is suitable for high-grade heat sources and has a wide range of power applications. Furthermore, it is commonly used in nuclear power, thermal power, solar power and the waste heat recovery of the gas turbine.

For the flue gas SCBC WHR of the marine diesel engines, Song established a Brayton cycle waste heat recovery system for turbocharged diesel engine and investigated the performance of a diesel engine integrated with the proposed system without considering the influence of the timing modulation parameters [13]. Bian proposed a model of a recompression SCBC and put forwards five kinds of control valves to investigate and compare the influence of different control valves on the open-loop dynamic performance of the system [14]. Feng proposed a combined SCBC with KALINA cycle power generation system WHR method for 8S90ME-C10.2 marine low-speed diesel engine and conducted the parameter analysis and optimization of the proposed cycle system [15]. EPS (ECHOGEN Power Systems), designed the EPS100 system for flue gas heat recovery from a ship’s main engine with an input power of 33.3 MW and a total output power of 8.6 MW, achieving a cycle efficiency of 24% [16]. GAO used a MAN B&W 6S50MC type low-speed two-stroke diesel engine as the research object, to study the diesel engine after exhaust modulation, through the waste heat boiler and power turbine to recover about 4% of the total energy, but did not consider the effect of exhaust modulation on the diesel engine performance comprehensively [17]. There are studies focused on optimizing the structure and the parameters of the S-CO_2_ Brayton cycle in the flue gas WHR from a ship’s main engine. Previous studies have not dealt with the influence of the timing modulation parameters on the performance of the WHR system. The modulation of the low-speed diesel engines with waste heat recovery systems is more complex and is a multi-parameter, multi-objective problem. On the premise of ensuring the diesel engine performance, the modulation of the S-CO_2_ Brayton cycle is used to complete the recovery of flue gas waste heat from the low-speed engine sufficiently. The practical significance of the modulation of the S-CO_2_ Brayton cycle is that the output power and efficiency of the system increased ultimately. There exists an optimal injection timing and exhaust timing that makes the system perform at its best from the perspective of thermodynamic performance. Thus, the goal of this article is to propose the laws of the timing modulation parameters for the marine low-speed diesel engine S-CO_2_ Brayton cycle. In conclusion, it is necessary to optimize the waste heat recovery system to improve fuel economy.

In this paper, the effect of low-speed engine timing on the performance of the S-CO_2_ Brayton cycle system for waste heat recovery is investigated by adjusting the timing (injection timing and exhaust timing) for a marine low-speed diesel engine with waste heat modulation. By establishing a one-dimensional model of the S-CO_2_ Brayton cycle system for flue gas waste heat recovery of the 6EX340EF marine low-speed diesel engine, the working process of the Brayton cycle was analyzed to reveal the variation pattern of the parameters and the net output work, thermal efficiency, and fuel consumption rate; the influence of injection timing and valve timing parameters on the performance of the main engine and the waste heat recovery is studied. The effect of injection timing and valve timing parameters on the performance of the main engine and the waste heat recovery system is investigated to determine the matching rules of the S-CO_2_ Brayton cycle parameters for flue gas waste heat recovery under different operating conditions. By optimizing the timing modulation parameters through MOGA and evaluating the flue gas waste heat recovery from the perspective of thermodynamic performance, the research on the performance modulation method of the S-CO_2_ Brayton cycle for flue gas waste heat in marine low-speed engines has been completed and can be extended to other engines.

## 2. Marine Low-Speed Engine Modeling and Validation

**Figure 1:**
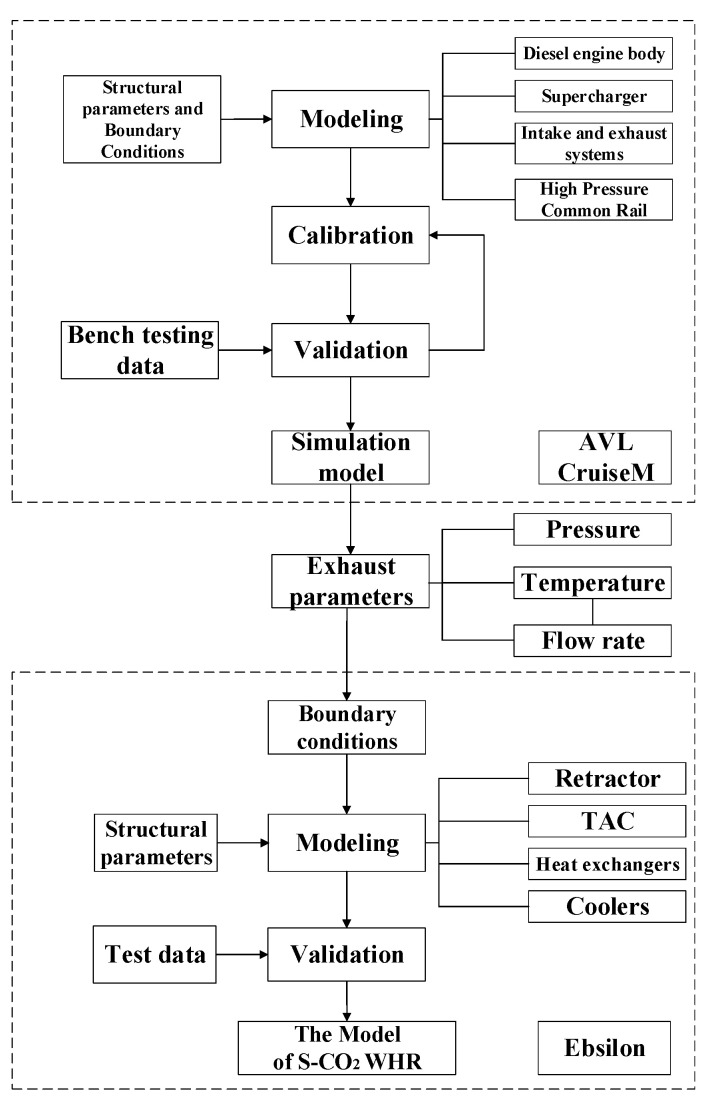
A 1D simulation flow chart.

The 6EX340EF marine low-speed diesel engine (hereinafter referred to as the low-speed engine) is used as the research object. A 1D simulation flow chart is shown in Figure 1. The simulation model is established in AVL Cruise M and verified the accuracy by the bench test data. The flue gas parameters of the low-speed engine model are used as input to the flue gas waste heat recovery system, and a simulation model of the S-CO_2_ Brayton cycle system for flue gas waste heat recovery of a low-speed engine is established in EBSILON. The matching law between different operating conditions of the marine low-speed engine and the parameters of the S-CO_2_ Brayton cycle for flue gas waste heat recovery is determined. The flue gas waste heat recovery is evaluated from the aspects of thermodynamic performance and energy saving and emission reduction efficiency.

### 2.1. Marine Low-Speed Diesel Engine Modeling

The main parameters of this low-speed diesel engine are shown in Table 1. The development of the model is carried out according to the operating principle of the low-speed diesel engine using a modular modelling approach.

**Figure 2:**
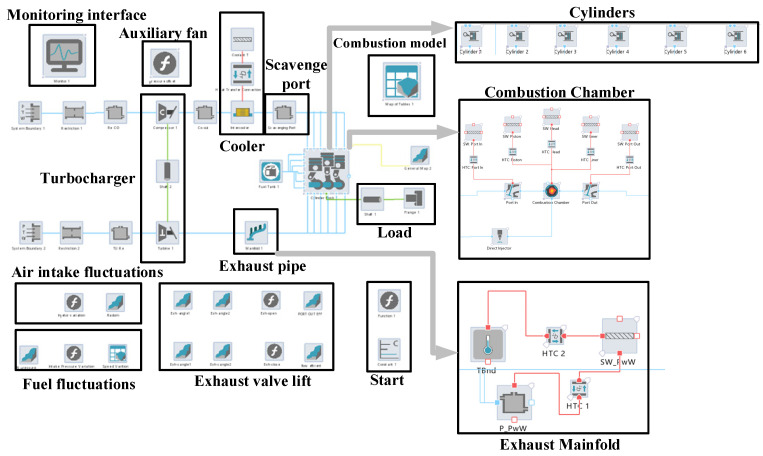
The 6EX340EF engine AVL Cruise M model.

The constructed real-time simulation model of the low-speed engine is shown in Figure 2, which mainly consists of modules such as the low-speed engine body, fuel system, intake and exhaust system (auxiliary fan, sweep box, exhaust pipe), intercooler and turbocharger [18]. The low-speed engine drives the compressor through the turbine to compress air into the sweep box, and through the sweep process, fresh gas is charged into the cylinder to push the exhaust gas out, and the high-temperature and high-pressure exhaust gas recharges the turbine for the next cycle [19].

### 2.2. The Algorithm Fitting for Marine Low-Speed Diesel Engine Model

For multi-objective function optimization computational processes, the design parameters and constraint variables can hardly present a linear, continuous, single-solution set relationship. In the practical development of the Cruise M model, to quickly solve the problems of the simulation model debugging in engineering practice and to make the model more similar to the real engine, this paper uses the cylinder pressure and the rate of heat release (ROHR) measured on the low-speed engine bench as the target curve, analyses the influence of the combustion model parameters on the simulation results, and adopts the MOGA algorithm to realize the automatic calibration of the model.

A genetic algorithm, which simulates the evolution of a biological process, is used to optimize the multi-objective function in order to efficiently find the balance between the various optimization objectives. The genetic algorithm allows for the inheritance and crossover of sample traits from the parent generation, while the sample traits from the offspring generation have a certain chance of mutation, and gradually approaches the optimal set of solutions by increasing the number of samples and iterations [20]. The MOGA algorithm is widely used in different optimization scenarios for its logical simplicity and computational efficiency, with high robustness and search efficiency, and the ability to avoid becoming trapped in a locally optimal solution set [21], and the MOGA algorithm parameters are set as shown in Table 2.

**Figure 3:**
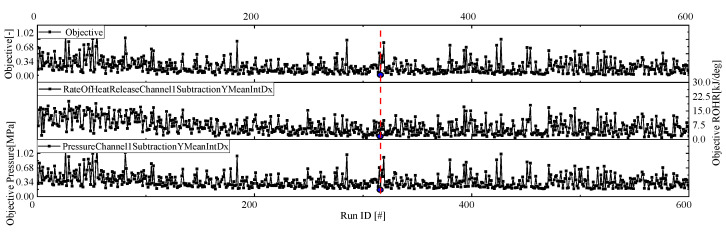

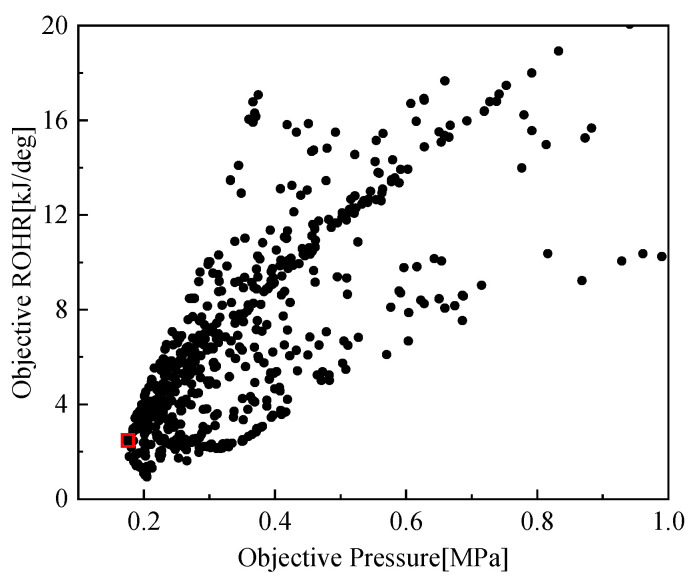
Pareto front surface of combustion parameters at 100% load.

The Pareto front surface of the combustion parameters at 100% load is shown in Figure 3, the total number of samples is 600, the horizontal coordinate is the absolute integral of the difference between the simulated cylinder pressure and the test value, and the vertical coordinate is the combustion heat release rate. The optimum combination of combustion parameters for 100% load is 361 and the error in the exothermic rate curve is within acceptable limits.

The optimum combustion parameters for each load were obtained by fitting the cylinder pressure curve and the ROHR curve with the MOGA algorithm, and the optimum combustion parameters for other loads are shown in Table 3. The optimum combustion parameter for 75% load was 529, 155 for 50% load and 520 for 25% load.

### 2.3. The Validation of Marine Low-Speed Diesel Engine Model

**Figure 4:**
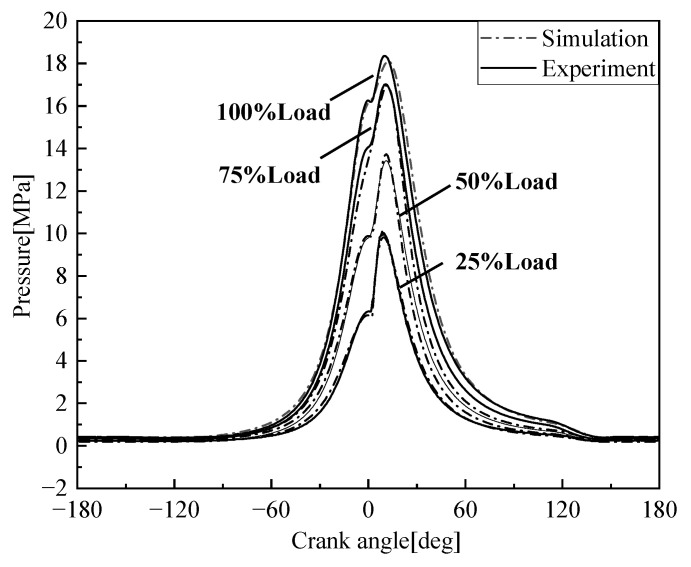
Comparison of 6EX340EF simulation and test cylinder pressure.

The marine low-speed diesel engine is operated according to the load characteristics. Figure 4 shows the comparison between the cylinder pressure simulation data and the test data at rated speed and different loads. The cylinder pressure simulation data and the test data fit well, the maximum error does not exceed 5%, so the model can better reflect the performance of the diesel engine.

**Figure 5:**
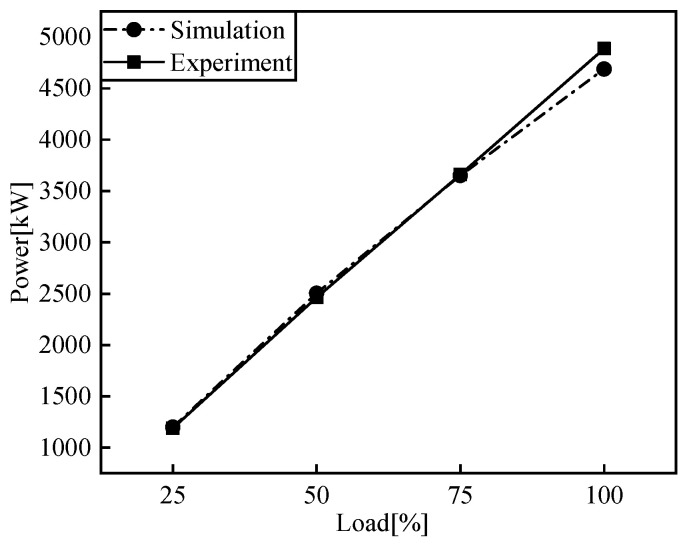
Comparison of output power.

**Figure 6:**
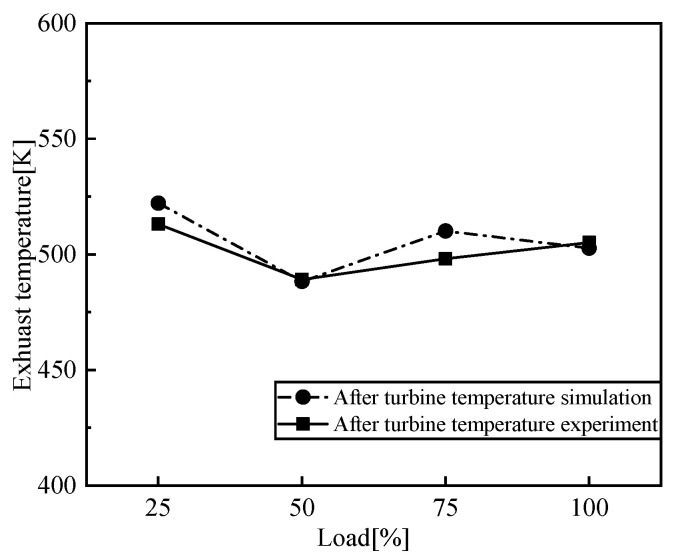
Comparison of exhaust temperature.

**Figure 7:**
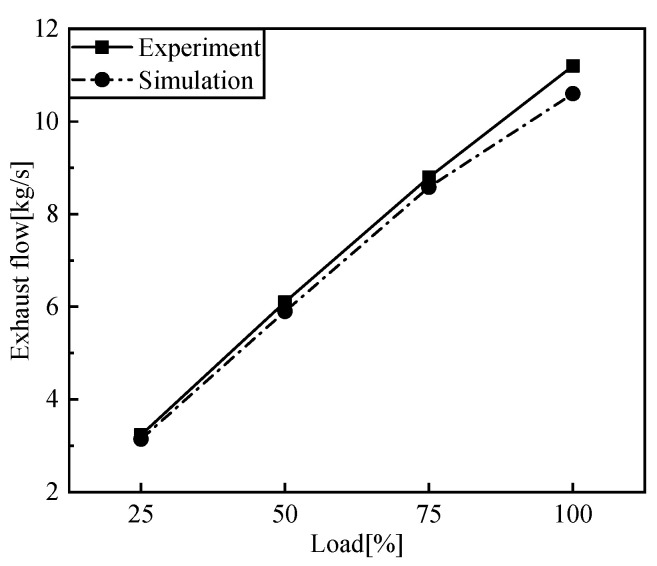
Comparison of exhaust flow.

**Figure 8:**
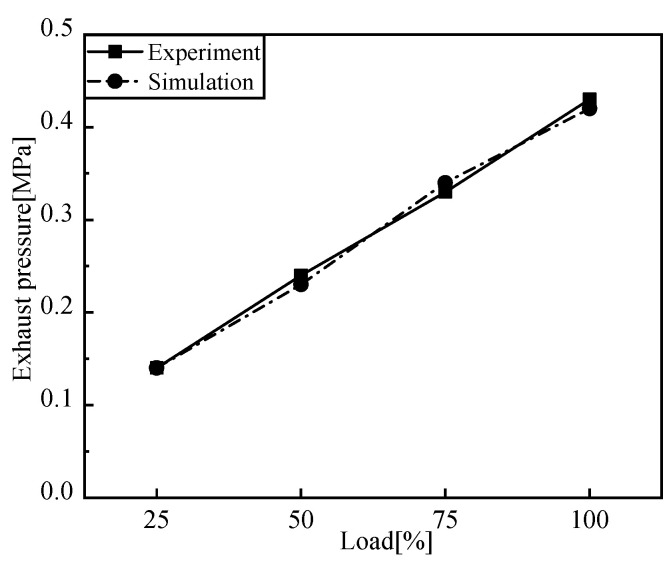
Comparison of exhaust pressure.

Figure 5, Figure 6, Figure 7 and Figure 8 show the comparison of the output power and exhaust parameters obtained from the real-time simulation model under different operating conditions with the bench test data. From Figure 8, we can see that the cylinder pressure calculated by the simulation model is in good agreement with the measured data, and the simulation model established for the low-speed engine can well characterize the cylinder pressure variation pattern. This is probably due to the use of a simplified heat transfer model in the in-cylinder heat transfer, where the wall temperature is set to a constant value, resulting in a generally large exhaust temperature. In reality, the instantaneous in-cylinder wall temperature should fluctuate and the distribution of the wall temperature is different.

From the above comparison of exhaust data, power and cylinder pressure at different loads with the measured data, it can be seen that the simulated data is in good agreement with the experimental data, within 5% error, and the developed simulation model of the low-speed engine can better reflect the operating conditions of the low-speed diesel engine under various operating conditions. The flue gas parameters under different working conditions are obtained as the input of the S-CO_2_ Brayton cycle system.

## 3. S-CO_2_ Brayton Cycle Waste Heat Recovery Model

### 3.1. Flue Gas Waste Heat Recovery Layout

This section may be divided by subheadings. It should provide a concise and precise description of the experimental results, their interpretation, as well as the experimental conclusions that can be drawn.

CO_2_ in the supercritical region has the physical characteristics of chemical stability, high thermal conductivity and small compression factor, small volume flow rate, and its low viscosity also reduces the friction loss of the working mass and consumes less compression work, thus enhancing the energy conversion efficiency of the Brayton cycle from the root [22]. It consists of four processes: isentropic compression by compressor, isobaric heat absorption by the ship’s main engine flue gas heat exchanger (hereafter referred to as heat exchanger), isentropic expansion by turbine and isobaric heat release by cooler.

The S-CO_2_ Brayton cycle has a high compressor power consumption due to the high turbine outlet temperature, while the cooler cools the mass to reduce the compressor power consumption, but it takes away the heat recovered from the system, making its cycle efficiency low. The system heats the mass back in the heat exchanger before it is compressed, and the recovered heat is used to heat the compressed mass, making the cycle efficiency increase. The S-CO_2_ Brayton cycle that only adds the reheat process is called the simple reheat Brayton cycle [23]. By building a simulation model of the S-CO_2_ Brayton cycle with different arrangement forms and comparing the cycle efficiency, it is found that the S-CO_2_ recompression Brayton cycle is more suitable for the flue gas waste heat recovery of the low-speed engine [24].

**Figure 9:**
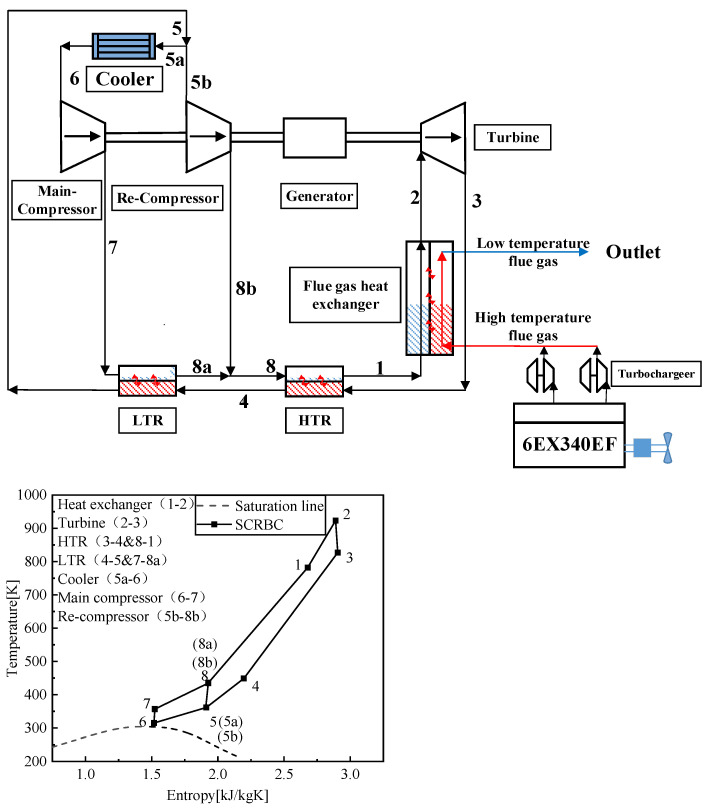
SCRBC system and working process.

Figure 9 shows the SCRBC system and its working process. The working medium absorbs heat from the flue gas in the flue gas heat exchanger of the engine, and becomes a supercritical working medium with high temperature and pressure through the process of isobaric heat absorption (1–2). After the turbine, the working medium performs isentropic expansion (2–3), and then enters HTR (high-temperature regenerator) and LTR (low-temperature regenerator) for heat recovery. The working medium after heating and compression increases the circulating efficiency (3–4, 4–5). After LTR, the working medium is shunt compressed, and isentropic compression (6–7, 5B–8B) is completed in the main compressor and the re-compressor, respectively. There is cooler in front of the main compressor to complete the intercooling process (5A–6) to reduce the power consumption of the compressor. The compressed working medium completes the heat recovery and confluence process (7–8A, 8–1) in HTR and LTR, and then enters the next cycle.

### 3.2. SCRBC Thermodynamic Model

The SCRBC model is established based on EBSILON platform to simulate the process and calculate the related parameters. The calculation assumptions are as follows:

- The system is in a stable state, and the change in kinetic energy is ignored;
- Isentropic efficiency is adopted for the turbines and compressors;
- Except the cooler, the heat transfer loss of the whole cycle and environment is ignored;
- The effectiveness of HTR and LTR are considered;
- The end difference at the cooler, regenerator and heat exchanger is considered;
- The parameters of each state point do not change with time. The thermodynamic model of each component of the cycle is shown in Table 4.

The physical parameters of CO_2_ in the vicinity of its critical point are difficult to determine and its nature has a significant impact on both the analysis and the equipment of the cycle. Given that the data in REFPROP software is fitted from experimental data, the critical point for supercritical fluids is measured accurately and the CO_2_ physical parameters are called from REFPROP 9.1 [25].

The nonlinear change in CO_2_ is the root of the pinching problem [26]. The main reason for the pinch point problem is the abrupt change in the specific heat capacity of CO_2_ during the heat exchange process and the proposed critical point of the mass in the heat exchanger, resulting in an abrupt change in the slope of the cold fluid in the Q–T graph of the heat exchanger [27]. The occurrence of pinch points in the Brayton cycle depends on the pressure of the fluid on both sides of the recuperator and the cold fluid inlet temperature. For the Brayton cycle, the higher the pressure of the hot fluid in the recuperator, the higher the pre-cooler inlet temperature threshold for pinch points in the recuperator; the higher the pressure of the cold fluid in the recuperator, the lower the pre-cooler outlet temperature threshold. For the ideal S-CO_2_ reheat cycle, the critical pre-cooler outlet temperature is linearly related to the turbine inlet pressure and turbine exhaust pressure.

(1) $$\left\{\begin{array}{c} \delta Q=-C_{P,hot}{\overset{\cdot }{m}}_{hot}\delta T_{hot}=C_{P,cold}{\overset{\cdot }{m}}_{cold}\delta T_{cold}\\ k_{hot}=-\frac{\delta T_{hot}}{\delta Q}=\frac{1}{C_{P,hot}{\overset{\cdot }{m}}_{hot}}\\ k_{hot}=\frac{\delta T_{cold}}{\delta Q}=\frac{1}{C_{P,cold}{\overset{\cdot }{m}}_{cold}} \end{array}\right.$$

where $k_{hot}$, $k_{cold}$ are the slopes of the discharge and suction lines, respectively.

To determine whether a proposed critical point occurs inside the heat exchanger, the inlet temperature of the cold fluid in the heat exchanger is compared with the magnitude of the proposed critical temperature, based on the Antonie equation describing the saturation temperature of the mass, the proposed critical temperature and the mass pressure, and the multivariable compression process:

(2) $$\left\{\begin{array}{c} \frac{T_{6}^{\kappa }}{P_{3}^{\kappa -1}}=\frac{T_{7}^{\kappa }}{P_{2}^{\kappa -1}}\\ \begin{array}{c} T_{7}=T_{6}{\left(\frac{P_{2}}{P_{3}}\right)}^{\frac{\kappa -1}{\kappa }}\\ T_{7}\geq T_{pseudo}=\frac{B}{A-\ln P}-C \end{array} \end{array}\right.$$

where, for the work substance CO_2_, *A* is taken as 9.64177, *B* as 1284.07 and *C* as 268.432.

(3) $$T_{6}\geq \left(\frac{B}{A-\ln P_{3}}-C\right)\times {\left(\frac{P_{3}}{P_{2}}\right)}^{\frac{\kappa -1}{\kappa }}$$

**Figure 10:**
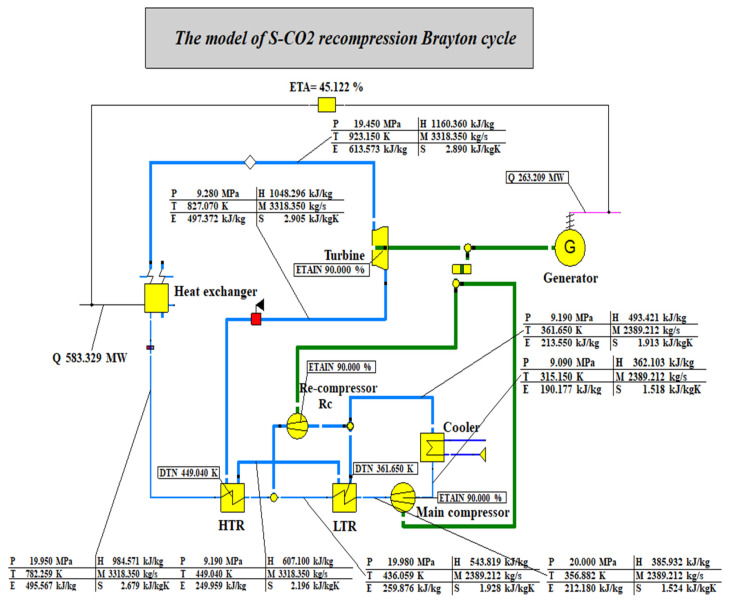
The model of S-CO_2_ recompression Brayton cycle.

The component parameters in the SCRBC were set and modelled [28]. Figure 10 shows the S-CO_2_ recompression Brayton cycle built on EBSILON platform. Table 5 compares the simulation with the test of the recompression cycle equipment.

Table 5 shows a comparison between the simulated and the experimental values of the recompression cycle equipment. It can be seen from the table that the simulated value calculated by the model is close to the test value in the Sandia laboratory, and the deviation is within 5%. Because the isentropic efficiency of the model turbine is set slightly lower, the turbine power is low, and the efficiency is 5.21% lower than the experimental value. It is considered that the established model is accurate and can be used for subsequent calculation.

### 3.3. Matching of WHR Parameters for Multiple Operating Conditions

**Figure 11:**
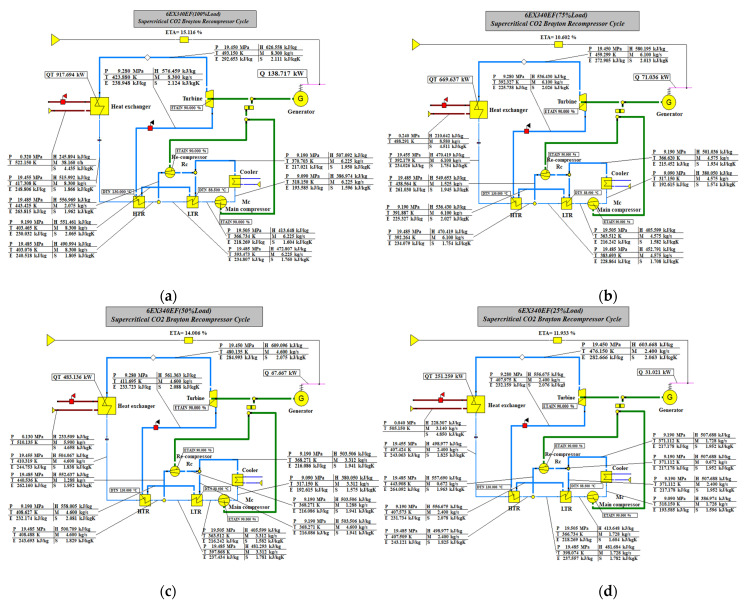
SCRBC for flue gas WHR. (**a**) 100% load, (**b**) 75% load, (**c**) 50% load, (**d**) 25% load.

The effect of each cycle parameter on the cycle efficiency is presented in the paper [23], where the effect of cycle parameters such as maximum cycle temperature, maximum cycle pressure, split ratio, compressor inlet pressure and compressor inlet temperature on the system efficiency is analyzed. As shown in Figure 11, the Brayton cycle for flue gas waste heat recovery and recompression at different loads is shown. The flue gas waste heat recovery at each load is shown in Table 6.

Table 6 shows the flue gas waste heat recovery at each load of the low-speed engine. As can be seen from the table, the total system efficiency of the flue gas from the low-speed engine at different operating conditions is improved to a certain extent after passing the SCRBC, and the Brayton cycle efficiency and net recovered work are strongly influenced by the exhaust gas temperature.

## 4. Timing Adjustment for Waste Heat Modulation

Low-speed engine waste heat modulation is generally carried out by adjusting the injection timing and exhaust timing of the diesel engine by adjusting the low-speed engine timing. The effects of different injection timings and exhaust timings on the performance of the low-speed engine and the effects of the flue gas waste heat recovery system are considered, and finally the appropriate timing of the low-speed engine is evaluated from the perspective of thermodynamics and emissions to achieve the residual heat modulation.

The effect of exhaust timing on the performance of the main engine is analyzed, and a model of the main engine’s flue gas waste heat recovery is built to analyze the effect of changes in the opening time of the exhaust valve on the performance of the waste heat recovery system through simulation. The impact of the change in exhaust valve opening time on the performance of the waste heat recovery system is analyzed through simulation results.

### 4.1. The Effect of Injection Timing on Residual Heat Modulation

**Figure 12:**
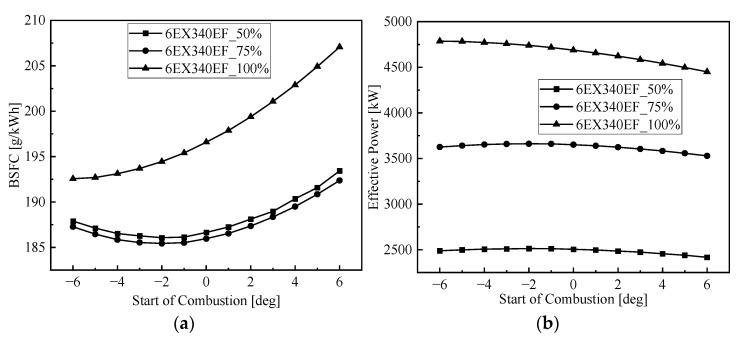

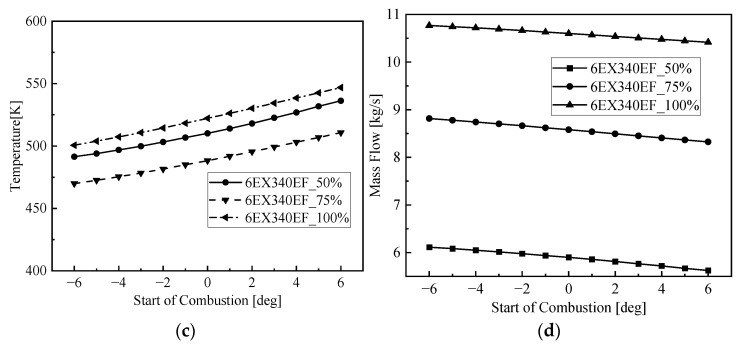
Effect of injection timing on the marine low-speed diesel engine. (**a**) Effect of injection timing on BSFC, (**b**) effect of injection timing on effective power, (**c**) effect of injection timing on temperature, (**d**) effect of injection timing on mass flow.

On the basis of the original injection timing, the injection timing was calculated in advance by 6 deg CA (crank angle) and delayed by 6 deg CA, with one data point calculated every 1 deg CA. The changes in exhaust temperature, exhaust flow and fuel consumption for the low-speed engine with different injection timings are shown in Figure 12. The effect of injection timing on WHR performance parameters was investigated by adjusting the injection timing.

From the effect of injection timing on the performance of the low-speed engine in Figure 12, it can be seen that as the injection timing is advanced, the fuel consumption rate and exhaust temperature are gradually reduced, which is because the injection timing is advanced, the fuller the oil and gas mixture in the cylinder, the more complete the combustion, the higher the cylinder pressure, and the output power increases. As the injection angle is delayed, the fuel cannot be burned in time near the upper stop, which will make the combustion of the main engine worse, the fuel consumption rate will rise and the exhaust temperature will increase. The output power of the low-speed engine with different working conditions increases with the increase in the injection advance angle first, when increased to a certain angle, the power no longer increases but gradually decreases.

**Figure 13:**
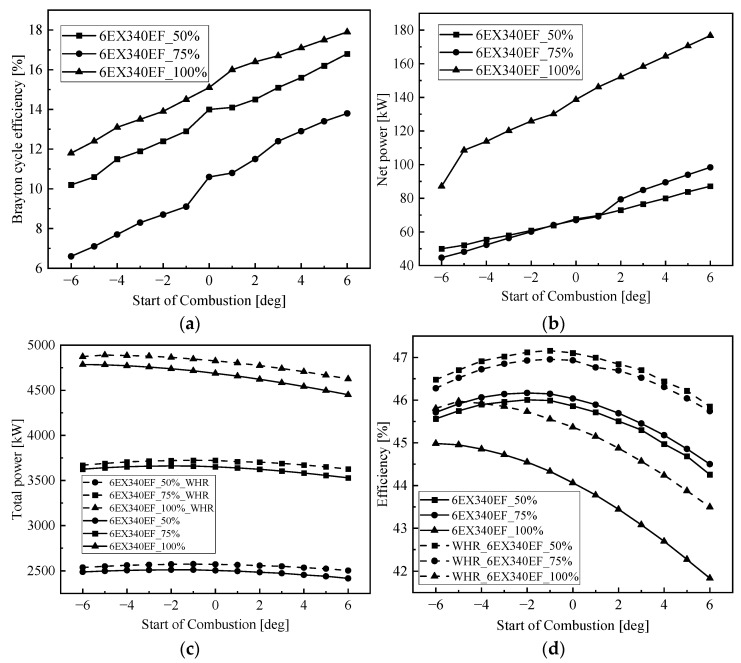
Effect of injection timing on residual heat modulation. (**a**) Effect of injection timing on Brayton efficiency, (**b**) effect of injection timing on net power, (**c**) effect of injection timing on total power, (**d**) effect of injection timing on efficiency.

Figure 13 shows the effect of injection timing on the system. From Figure 13a,b, it can be seen that although the net cycle recovery work and Brayton cycle efficiency increase with the delay of injection timing, the output work of the low-speed engine decreases with the delay of injection timing, which is because the net cycle recovery work and Brayton cycle efficiency are greatly affected by the exhaust temperature, while the exhaust temperature is negatively related to the output power. From Figure 13c,d, we can see that, after the addition of the S-CO_2_ Brayton cycle system for flue gas waste heat recovery of the low-speed engine, the total output power and cycle efficiency of the low-speed engine at all loads are significantly improved; the change pattern of the system with the injection timing is not the same as that of the original engine, which mainly depends on the influence of the injection timing on the performance of the low-speed engine and the performance of the Brayton cycle. Taking this into account, the total output power at 100% load reaches a maximum of 4891 kW at an injection timing of −5 deg CA, the total output power at 75% load reaches a maximum of 3724 kW at an injection timing of −1 deg CA and the total output power at 50% load reaches a maximum of 2576 kW at an injection timing of −1 deg CA.

### 4.2. The Effect of Exhaust Timing on Residual Heat Modulation

**Figure 14:**
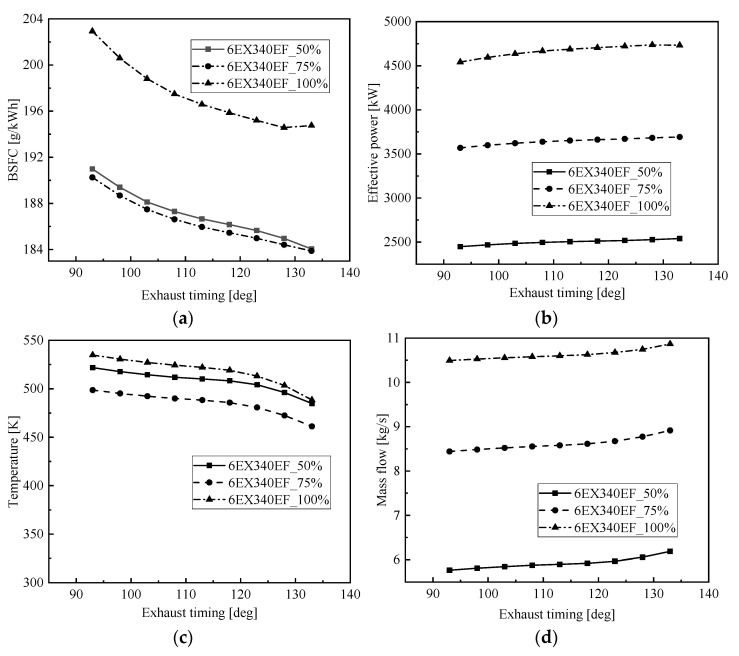
Effect of exhaust timing on the marine low-speed diesel engine. (**a**) Effect of exhaust timing on BSFC, (**b**) effect of exhaust timing on effective power, (**c**) effect of exhaust timing on temperature, (**d**) effect of exhaust timing on mass flow.

The opening time of the exhaust valve has an important influence on the sweeping process of the low-speed, two-stroke diesel engine, which directly affects the performance parameters of the main engine [29]. To study the effect of different exhaust timings on the low-speed engine, calculations were made on the basis of the opening moment of the exhaust valve of the original engine, 20 deg CA ahead to 20 deg CA behind, and the change pattern of exhaust temperature, exhaust flow and fuel consumption of the main engine under different exhaust timing at common load was obtained as shown in Figure 14. From the figure, it can be seen that the flue gas temperature and fuel consumption rate of the low-speed engine decrease as the opening moment of the exhaust valve is delayed, because at this time the burst pressure in the cylinder increases and the work performed by expansion increases, which eventually leads to an increase in the effective power and a decrease in the exhaust temperature and fuel consumption rate.

**Figure 15:**
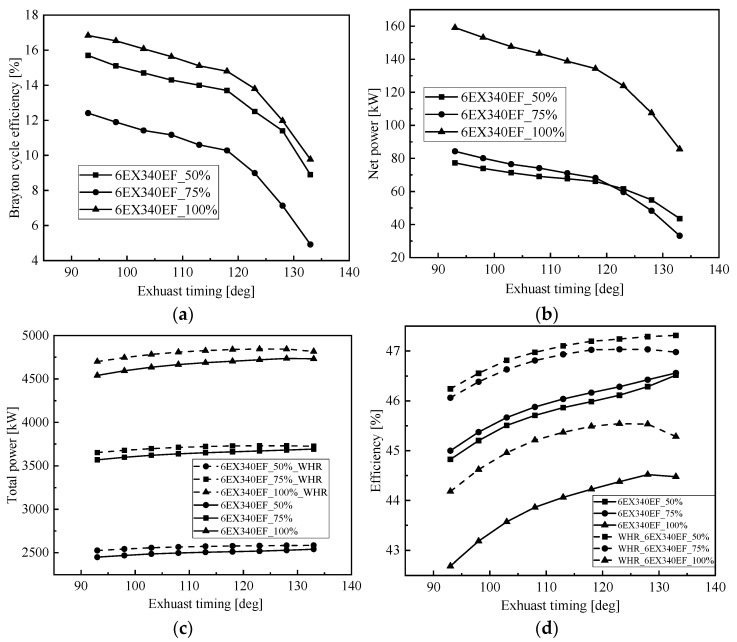
Effect of exhaust timing on residual heat modulation. (**a**) Effect of exhaust timing on Brayton efficiency, (**b**) effect of exhaust timing on net power, (**c**) effect of exhaust timing on total power, (**d**) effect of exhaust timing on efficiency.

Figure 15 shows the effect of exhaust timing on waste heat modulation, from the figure, it can be seen that although the net cycle recovery work and Brayton cycle efficiency decrease with the delay of exhaust timing, the output work of the low-speed engine increases with the delay of exhaust timing; after adding the flue gas waste heat recovery S-CO_2_ Brayton cycle system, the total output power and cycle efficiency of each load of the marine low-speed engine are significantly improved; the system changes with the exhaust timing The change pattern with exhaust timing is not the same as that of the original engine, which mainly depends on the influence of exhaust timing on the performance of the low-speed engine and the performance of the Brayton cycle. The total output power for 100% of the load reaches a maximum of 4845 kW at 128 deg CA, for 75% of the load reaches a maximum of 3730 kW at 128 deg CA and for 50% of the load reaches a maximum of 2584 kW at 133 deg CA.

## 5. S-CO_2_ Waste Heat Recovery Performance Evaluation

### 5.1. Optimized Timing Modulation Based on MOGA

**Figure 16:**
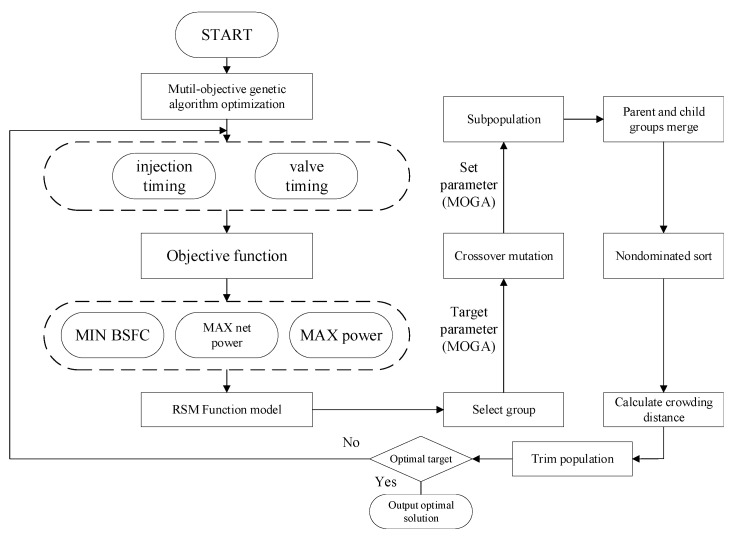
Flow chart for multi-objective optimization.

In terms of the system as a whole, although the engine output can be increased by adjusting the timing, the exhaust temperature will be reduced, which in turn significantly reduces the net recovered work from waste heat recovery. For multi-objective optimization problems, the objective is to find all possible optimal solutions by means of a multi-objective genetic algorithm, and the MOGA can find the optimal solution by means of a Pareto front surface [30]. From a thermodynamic perspective, the objective of this paper is to optimize the injection and exhaust timing by MOGA to achieve the highest effective power, the maximum net flue gas recovery work and the lowest fuel consumption rate, with the process shown in Figure 16.

The optimal Latin hypercube design has very good space filling and equilibrium, creating a standard second order least squares polynomial response surface to approximate the test points [22]. The response surface methodology (RSM) considers experimental random errors and fits complex unknown functional relationships to simple primary or quadratic polynomial models. The calculation is relatively simple and the resulting predictive model is continuous, making it well suited to RSM for the analysis of BSFC, power and net flue gas recovery work. The expression for a multiple quadratic regression mathematical model [31] based on sample points is as follows:

(4) $$y=\beta_{0}+\sum_{i=1}^{k}\beta_{i}x_{i}+\sum \sum_{i<j}\beta_{ij}x_{i}x_{j}+\sum_{i=1}^{k}\beta_{ii}x_{i}^{2}+\varepsilon$$

where y is the response; $x_{i}$ is the value of the factor, $x_{1}$ injection timing, $x_{2}$ exhaust timing; $\beta_{0}$, $\beta_{i}$, $\beta_{ii}$ and $\beta_{ij}$ is the regression coefficient; ε is the error.

The multi-objective genetic algorithm used is able to identify the optimal value of the target, and the MOGA sets the parameters: number of samples 600, evolutionary generation 10, crossover factor 0.5 and mutation factor 0.5. The genetic algorithm also introduces systematic constraints to ensure that the algorithm converges to a feasible solution [32]. The constraints are as follows:

(5) $$\left\{\begin{array}{c} x_{1}\in X_{1}\subseteq \left[-6,6\right]\\ x_{2}\in X_{2}\subseteq \left[-20,20\right] \end{array}\right.$$

The targets are as follows:

(6) $$\left\{\begin{array}{c} \min\;y_{1}\left(BSFC\right)\\ \max\;y_{2}\left(Power\right)\\ \max\;y_{2}\left(Net\;Power\right) \end{array}\right.$$

**Figure 17:**
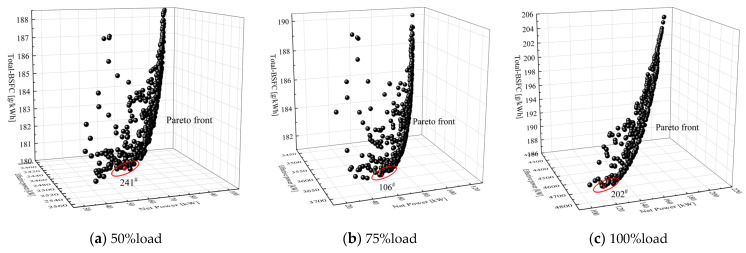
MOGA pareto front surface for operating conditions. (**a**) 50% load, (**b**) 75% load, (**c**) 100% load.

As can be seen from the Pareto front surface plots for each load, as shown in Figure 17, the MOGA used in this paper is applicable to the problem under study, i.e., achieving the objectives of maximum power, maximum net power and minimum *BSFC*. The red dots indicate that they are Pareto-optimal solutions and all Pareto-optimal solutions form the Pareto-optimal solution set.

Table 7 shows the MOGA optimization results for this system at 100% load conditions, with the top five waste heat timing modulation schemes given in the table. At this load, although the 202 scheme is not the largest waste heat recovery work, the overall system efficiency reaches a maximum value of 46.13% and the fuel consumption rate reaches a minimum of 187.78 g/kWh in terms of the system as a whole.

The optimum points of the modulation of the flue gas waste heat recovery timing at each load of the marine low-speed engine after optimization by the MOGA algorithm are shown in Table 8, where the total system efficiency is significantly improved at the corresponding optimized timing point at each load, and the maximum value.

### 5.2. Thermodynamic Evaluation

**Figure 18:**
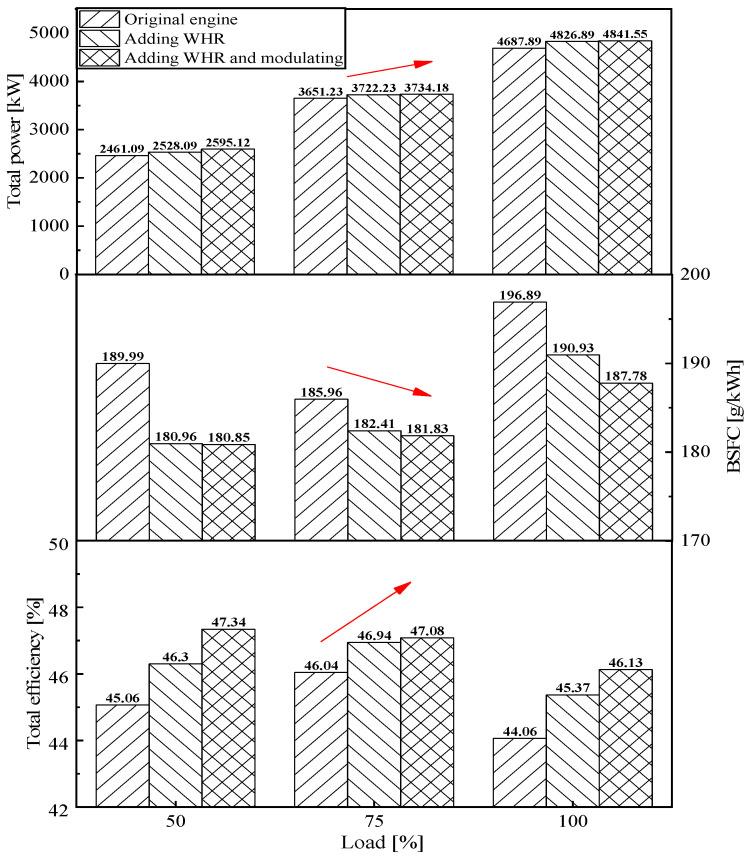
Comparison of the thermodynamic properties under various loads.

From the thermodynamic analysis of the original engine, the SCRBC system with the addition of waste heat recovery and the SCRBC system with the addition of waste heat modulation in Figure 18, it can be seen that the addition of the SCRBC system with waste heat recovery results in an increase in total power and efficiency and a decrease in fuel consumption rate. The SCRBC with waste heat modulation will further increase the total power and efficiency, which in turn brings about a reduction in fuel consumption rate. The efficiency of the SCRBC system with the addition of waste heat recovery increases by 1.24%, 0.90% and 1.31% at 50%, 75% and 100%, respectively, while the efficiency of the SCRBC system with the addition of waste heat modulation increases by 2.28%, 1.04% and 2.07% at 50%, 75% and 100%, respectively. The reason for the lowest Brayton cycle efficiency under 75% load is that the efficiency is most sensitive to the parameter of maximum cycle temperature, which depends on the flue gas temperature. Furthermore, the lowest flue gas temperature occurred at 75% load in low-speed engine bench test. The maximum fuel consumption rate reduction of 9.11 g/kWh occurred for the SCRBC system with waste heat modulation at 100% load.

### 5.3. Emissivity Evaluation

The energy saving and emission reduction benefits include fuel consumption reduction and annual CO_2_ reduction, reflecting the energy saving and emission reduction benefits of the system retrofit compared to the original engine.

Fuel consumption rate for low-speed engine:

(7) $$BSFC_{DE}=m_{fuel}/W_{DE}$$

where $m_{fuel}$ and $W_{DE}$ are the hourly fuel consumption and power rating of the low-speed engine.

Low-speed diesel engine-SCRBC combined cycle fuel consumption rate:

(8) $$BSFC_{DE\text{-}SRCBC}=m_{fuel}/(W_{DE}+P_{net})$$

Combined low-speed engine-SCRBC fuel consumption rate after timing modulation:

(9) $$BSFC_{DE\text{-}MOGA}=m_{fuel}/(W_{DE}+P_{net\text{-}MOGA})$$

Annual CO_2_ reductions from combined low-speed engine-SCRBC:

(10) $$R_{CO_{2}\text{-}DE\text{-}SCRBC}=(P_{net}/W_{DE})m_{fuel}h_{full\text{-}load}f_{CO_{2}}$$

Annual CO_2_ emission reduction from combined low-speed engine-SCRBC after timing modulation:

(11) $$R_{CO_{2}\text{-}DE\text{-}MOGA}=(P_{net\text{-}MOGA}/W_{DE})m_{fuel}h_{full\text{-}load}f_{CO_{2}}$$

where $h_{full\text{-}load}$ is the full year operating time of the system, set to 8200 h; $f_{CO_{2}}$ is the CO_2_ emission factor for the complete combustion of 1 kg of diesel, taken as 3.1863 [33].

The fuel consumption rates and annual CO_2_ emission reductions for each load of the low-speed engine are shown in Table 9, with the maximum annual CO_2_ emission reduction of 748.51 × 10^3^ kg·a^−1^ occurring in the SCRBC system with the addition of waste heat modulation at 50% load.

### 5.4. Exergy Analysis

The exergy analysis of SCRBC system for low-speed engine flue gas waste heat recovery aims to evaluate the weak points in the energy conversion process by calculating the exergy loss and exergy loss efficiency of the system and its components. The system is optimized and analyzed to ultimately improve energy utilization [34].

**Figure 19:**
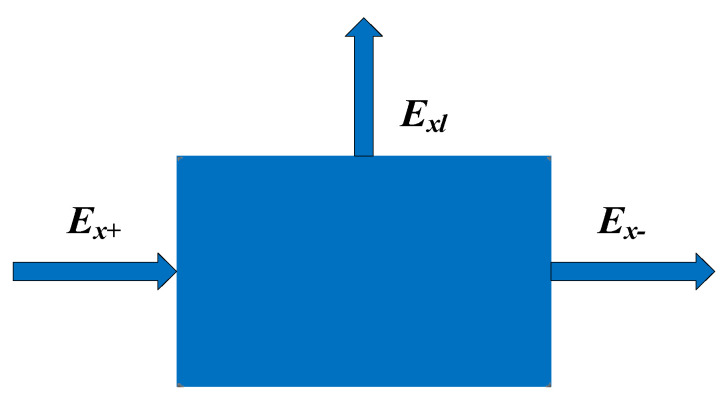
The grey box model for exergy analysis.

A grey-box model using the exergy analysis is shown in Figure 19, introducing three variables the supplying exergy ($E_{X+}$), the effective exergy ($E_{X-}$), and the total exergy loss ($E_{X,L}$), such that any components of the system can be represented as a network unit consisting of these three variables [35].

Balanced equations of the grey box model for the exergy analysis:

(12) $$E_{X+}-E_{X-}=E_{X,L}$$

(13) $$\eta_{exe}=\frac{E_{X-}}{E_{X+}}=1-\frac{E_{X,L}}{E_{X+}}$$

**Figure 20:**
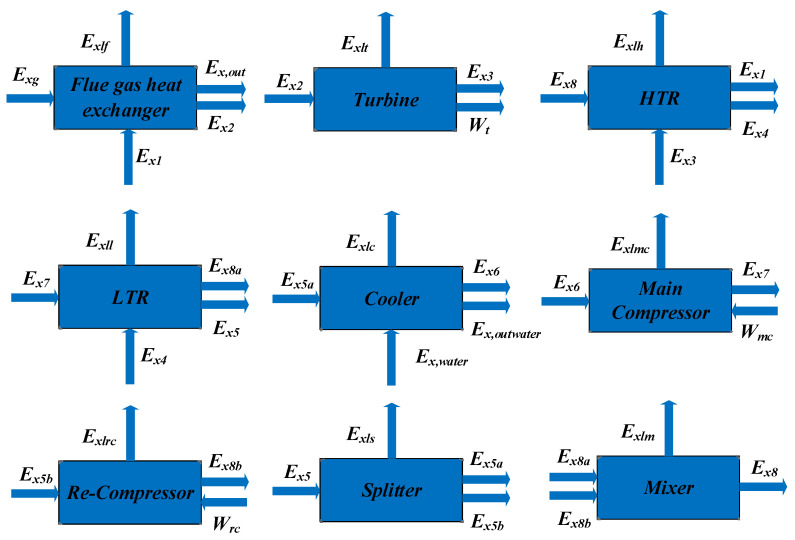
Exergy analysis of the SCRBC components.

The grey box model of the SCRBC components for the exergy analysis is shown in Figure 20, where the main components are the Flue gas heat exchanger, Turbine, HTR, LTR, Cooler, Main compressor, Re-compressor, Splitter and Mixer. The analysis of the model’s exergy loss and exergy efficiency are shown in Table 10, and there are also exergy flow links between the SCRBC components.

The exergy analysis is concerned with the quantity and quality of the energy conversion process, and can be used to determine the exergy loss and energy conversion weaknesses of each part of the thermal system [36].

**Figure 21:**
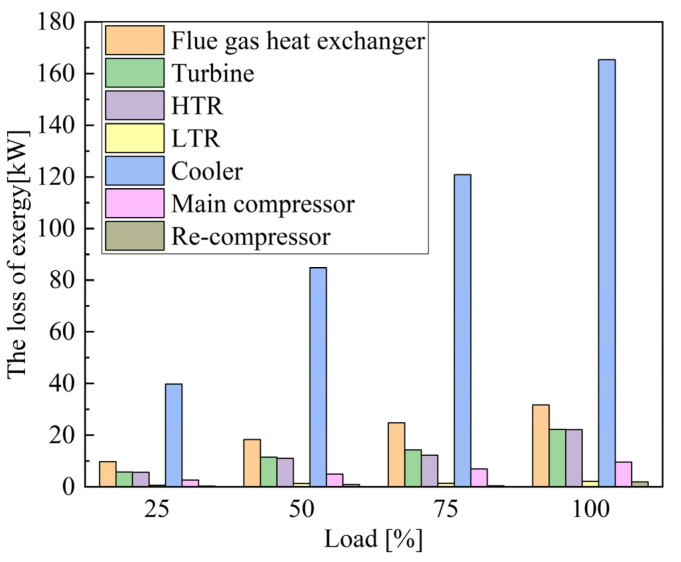
Exergy loss of the SCRBC.

**Figure 22:**
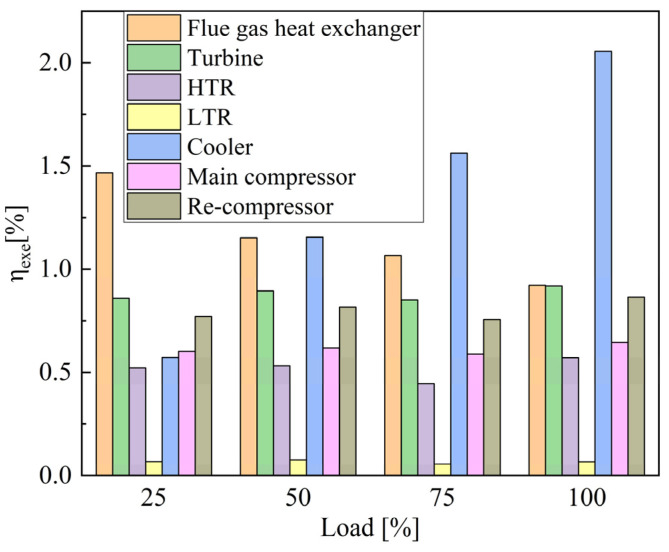
Exergy loss efficiency of the SCRBC.

Figure 21 and Figure 22 show the exergy loss and exergy loss efficiency of the SCRBC system under different flue gas loads. Under 100% load, the cooler has the largest system exergy loss of 165 kW, with the exergy loss efficiency of 2.06%. From the overall view of the system, the energy input of the system comes from the flue gas waste heat, and the output is the net output work, the energy taken away by the cooling water and the energy loss of various components, of which the energy taken away by the cooling water is the highest. The exergy losses of flue gas heat exchanger are also 32 kW, with the exergy loss efficiency of 0.92%. Further optimization of the SCRBC system should first focus on improving these two components.

## 6. Conclusions

(1) With the structural parameters of 6EX340EF marine low-speed diesel engine, the low-speed engine model was established in AVL Cruise M and verified by the bench test data. The optimum combustion parameters for each load were obtained by fitting the cylinder pressure curve and the ROHR curve with the MOGA algorithm. A one-dimensional simulation model of the S-CO_2_ recompression Brayton cycle system for the low-speed engine flue gas waste heat recovery was developed in EBSILON, and the accuracy of the model was verified by SANDIA experimental data. On this basis, the effects of injection timing and valve timing parameters on the comprehensive performance of the main engine and the waste heat recovery system were investigated.
(2) By optimizing the timing modulation parameters through MOGA and evaluating the flue gas waste heat recovery from the perspective of thermodynamic performance, the research on the performance modulation method of the S-CO_2_ Brayton cycle for flue gas waste heat in marine low-speed engines has been completed. The SCRBC with waste heat modulation will further increase the total power and efficiency, which in turn brings about a reduction in fuel consumption rate. The efficiency of the SCRBC system with the addition of waste heat recovery increases by 1.24%, 0.90% and 1.31% at 50%, 75% and 100%, respectively, while the efficiency of the SCRBC system with the addition of waste heat modulation increases by 2.28%, 1.04% and 2.07% at 50%, 75% and 100%, respectively. The SCRBC system with the waste heat modulation had annual CO_2_ emission reductions for each load of the low-speed engine, and the maximum annual CO_2_ emission reduction of 748.51 × 10^3^ kg·a^−1^ occurred at 50% load.
(3) With the exergy analysis, the cooler has the largest system exergy loss of 165 kW, with the exergy loss efficiency of 2.06% under 100% load. The second is flue gas heat exchanger with the exergy loss of 165 kW, and the exergy loss efficiency of 2.06%. Further optimization of the SCRBC system should first focus on improving these two components.
(4) The evaluation of the low-speed engine flue gas waste heat recovery from the perspective of thermodynamic performance and energy saving and emission reduction efficiency was carried out. The research on the performance modulation method of S-CO_2_ Brayton cycle for flue gas waste heat in marine low-speed engine has been completed, which further improves the efficiency of the system and can be extended to other engines.

## Figures and Tables

**Table 1:** Low-speed engine technical parameters.

Parameters	Unit	Values
Bore	[mm]	340
Power	[kW]	4700
Rated speed	[r/min]	157
Compression ratio	[-]	20.5
Fire order	[-]	1-6-2-4-3-5
Cylinder	[-]	6
Rod	[mm]	1600 mm
Stroke	[mm]	1600 mm
Fuel injection	[-]	High pressure common rail (HPCR)
Exhaust valve drive	[-]	Electrical control
Main forms	[-]	Turbocharged, Intercooled, DC scavenging, Inline arrangement

**Table 2:** Properties for the MOGA algorithm.

Name	Value
Optimization	MOGA
Number of Generations	10
Population Size	50
Crossover Probability	0.5
Distribution for Crossover Probability	10
Mutation Probability	0.5
Distribution for Mutation Probability	10

**Table 3:** Optimization points for combustion parameters at various loads.

Load	Run ID	Start of Combustion [Deg]	Vibe Shape Parameter M[-]	Combustion Duration [Deg]	Obj_Pressure_ [MPa]	Obj_ROHR_ [kJ/Deg]
100%	361	2.0694	0.6639	58.2864	0.1736	2.5597
	546	2.7279	0.5854	58.4207	0.1761	2.3935
	584	2.0015	0.5910	58.4208	0.1776	1.7935
75%	529	−9.6757	3.0558	34.9973	0.2264	3.0055
	19	−9.5773	3.0715	33.8959	0.2301	3.2137
	173	−9.5773	3.2351	33.8959	0.2302	3.2483
50%	155	0.7546	1.2246	22.0988	0.1732	2.8007
	67	1.5750	0.7428	25.6988	0.1740	2.8501
	279	−1.8556	1.8593	23.2302	0.1753	2.8574
25%	520	2.5750	0.0048	45.0227	0.1231	1.8271
	459	1.7101	0.1378	40.5455	0.1233	1.6170
	359	1.7485	0.1378	38.8616	0.1234	1.7204

**Table 4:** Thermodynamic model of the components.

Components	Formula
Turbine	$W_{T}=h_{2}-h_{3}$ $\eta_{T,S}=\frac{h_{2}-h_{3}}{h_{2}-h_{3,S}}$
Main compressor	$W_{MC}=\left(1-r\right)\left(h_{7}-h_{6}\right)$ $\eta_{MC,S}=\frac{h_{7,S}-h_{6}}{h_{7}-h_{6}}$
Re-compressor	$W_{RC}=r\left(h_{8b}-h_{5b}\right)$ $\eta_{RC,S}=\frac{h_{8b,S}-h_{5b}}{h_{8b}-h_{5b}}$
Heat exchanger	$q_{Heater}=h_{2}-h_{1}$
HTR	$h_{3}-h_{4}=h_{1}-\left[rh_{8b}+\left(1-r\right)h_{8a}\right]$
LTR	$h_{4}-h_{5}=h_{8a}-h_{7}$
Cooler	$q_{Cooler}=\Delta h_{Cooler}=\left(1-r\right)\left(h_{6}-h_{5a}\right)$
Overall	$\eta_{Total}=\frac{\left(1-r\right)\left(\Delta h_{T}-\Delta h_{MC}\right)+r\left(\Delta h_{T}-\Delta h_{RC}\right)}{\left(1-r\right)\left(\Delta h_{T}-\Delta h_{MC}+\Delta h_{Cooler}\right)+r\left(\Delta h_{T}-\Delta h_{RC}\right)}$

In the table, *h*_i_ is the enthalpy of each measurement point, *r* is the shunt coefficient, Δ*h_MC_* is the enthalpy difference between the outlet and the inlet of the main compressor, Δ*h_RC_* is the enthalpy difference between the outlet and the inlet of the re-compressor, Δ*h_Heater_* is the enthalpy difference between the outlet and the inlet of the heat exchanger, Δ*h_T_* is the enthalpy difference between the inlet and the outlet of the turbine.

**Table 5:** Comparison of simulated and experimental values of recompression equipment.

Power [MW]	Sandia	Simulation	Deviation [%]
Cooler	315.5	313.75	0.55
Main compressor	56.30	57.51	2.2
Re-compressor	45.89	45.81	0.17
LTR	375.00	377.23	0.59
HTR	1452.40	1466.11	0.94
Heat exchanger	600.00	583.33	2.78
Turbine	388.00	371.12	4.35
Efficiency [%]	47.60	45.12	5.21

**Table 6:** Flue gas WHR under various loads.

Load [%]	Mass Flow [kg/s]	Exhaust Temperature [K]	Enthalpy [kJ/kg]	Entropy [kJ/kgK]	Brayton Efficiency [%]	Recovery Power [kW]	Efficiency Increase [%]
100	10.60	513	246.12	4.53	15.12	139	1.31
75	8.58	489	210.65	4.51	10.60	71	0.90
50	5.90	499	233.51	4.66	14.01	67	1.24
25	3.14	505	228.31	4.85	11.93	31	1.05

**Table 7:** MOGA optimization results at 100% load.

Run ID	Injection Timing [deg CA]	Exhaust Timing [deg CA]	Net Power [kW]	Output Power [kW]	Total Efficiency [%]	BCFS [g/kWh]
202	−4.07	116.79	112.52	4795.42	46.13	187.78
121	−2.94	114.49	121.60	4785.71	46.13	187.80
56	−3.73	116.65	114.69	4792.09	46.12	187.82
48	−4.55	114.43	111.73	4794.70	46.12	187.84
113	−4.32	114.43	112.93	4792.61	46.11	187.87

**Table 8:** The timing residual heat modulation for low-speed engine.

Load [%]	Injection Timing [deg CA]	Exhaust Timing [deg CA]	Net Power [kW]	Output Power [kW]	Total Efficiency [%]	BCFS [g/kWh]
100	−4.07	116.79	112.52	4795.42	46.13	187.78
75	−1.19	124.18	52.52	3681.66	47.08	181.83
50	−1.30	132.98	37.79	2547.78	47.34	180.85

**Table 9:** Annual CO_2_ emission reductions of the low-speed engine.

Load [%]	*BSFC_DE_* [g/kWh]	*BSFC_DE-SCRBC_* [g/kWh]	*BSFC_DE-MOGA_* [g/kWh]	*R*_*CO*_2_*-DE-SRCBC*_ [10^3^ kg·a^−1^]	*R*_*CO*_2_*-DE-MOGA*_ [10^3^ kg·a^−1^]
100	196.89	190.93	187.78	326.37	652.89
75	185.96	182.41	181.83	385.87	450.82
50	189.99	180.96	180.85	677.10	748.51

**Table 10:** Exergy analysis of the SCRBC components.

Components	Exergy Loss	*η_xl_*
Turbine	$E_{xlt}=E_{x2}-E_{x3}-W_{t}$	$\eta_{xlt}=\frac{E_{xlt}}{E_{2}}$
Main compressor	$E_{xlmc}=E_{x6}+W_{mc}-E_{x7}$	$\eta_{xlmc}=\frac{E_{xlmc}}{E_{x6}+W_{mc}}$
Re-compressor	$E_{xlrc}=E_{x5b}+W_{rc}-E_{x8b}$	$\eta_{xlrc}=\frac{E_{xlrc}}{E_{x5b}+W_{rc}}$
Heat exchanger	$E_{xlf}=E_{xg}+E_{x1}-E_{x2}-E_{x,out}$	$\eta_{xlf}=\frac{E_{xlf}}{E_{xg}+E_{x1}}$
HTR	$E_{xlh}=E_{x8}+E_{x3}-E_{x4}-E_{x1}$	$\eta_{xlh}=\frac{E_{xlh}}{E_{x8}+E_{x3}}$
LTR	$E_{xll}=E_{x7}+E_{x4}-E_{x8a}-E_{x5}$	$\eta_{\mathrm{xll}}=\frac{E_{xll}}{E_{x7}+E_{x4}}$
Cooler	$E_{xlc}=E_{x5a}+E_{x,water}-E_{x6}-E_{x,outwater}$	$\eta_{xlc}=\frac{E_{xlc}}{E_{x5a}+E_{x,water}}$

## Data Availability

Not applicable.

## Nomenclature

**Abbreviations**	
IMO	International Maritime Organization
WHR	Waste Heat Recovery
EEDI	Energy Efficiency Design Index
PT	Power Turbine
ET	Electrical Turbine
TEG	Thermoelectric Generator
ORC	Organic Rankine Cycle
SCBC	Supercritical Carbon Dioxide Brayton Cycle
S-CO_2_	Supercritical Carbon Dioxide
SCRBC	S-CO_2_ Recompression Brayton Cycle
MOGA	Multi-objective Genetic Algorithm
HPCR	High-Pressure Common Rail
ROHR	Rate of Heat Release
LTR	Low-Temperature Recuperator
HTR	High-Temperature Recuperator
BSFC	Brake Specific Fuel Consumption
CA	Crank Angle
RSM	Response Surface Methodology
Subscripts and superscripts	
T	Turbine
MC	Main Compressor
RC	Re-compressor
Heater	Heat Exchanger
Cooler	Cooler
LTR	Low-Temperature Recuperator
HTR	High-Temperature Recuperator
fuel	Diesel Fuel
*X*+	The Supplying Exergy
*X*−	The Effective Exergy
*X,L*	The Total Exergy Loss
*exe*	Exergy
*xlf*	Exergy Loss of Flue Gas Heat Exchanger
*xlt*	Exergy Loss of Turbine
*xlh*	Exergy Loss of HTR
*xll*	Exergy Loss of LTR
*xlc*	Exergy Loss of Cooler
*xlmc*	Exergy Loss of Main Compressor
*xlrc*	Exergy Loss of Re-compressor
*xls*	Exergy Loss of Splitter
*xlm*	Exergy Loss of Mixer
1, …8	Thermodynamic State Points (Figure 9)
**Symbols**	
*P*	Pressure (MPa)
*E*	Specific exergy (kJ/kg)
*q*	Thermal energy (kW)
*S*	Entropy (kJ/(kg·K))
*T*	Temperature (K or °C)
*W*	Work Down, Power (kW)
*h*	Specific Enthalpy (kJ/kg)
*η*	Efficiency (%)
*r*	Split ratio (-)
*k*	The Slope of (-)
*C_P_*	Specific Heat Capacity (kJ/(kg·K))
*m*	Mass Flow Rate (kg/s)
*κ*	Polytropic Coefficient
*x*	The Value of the Factor
*β*	The Regression Coefficient
ε	The Error
*R*	Annual CO_2_ Emission Reduction
*f*	The CO_2_ Emission Factor
